# Enriched Environment Promotes Cognitive Function Recovery following Cerebral Ischemic Injury via Upregulating GABAergic and Glutamatergic Systems in the Contralateral Hippocampus

**DOI:** 10.1155/2020/8850119

**Published:** 2020-12-15

**Authors:** Yuyang Wang, Junfa Wu, Hongyu Xie, Liping Wang, Chuanjie Wang, Yi Wu

**Affiliations:** ^1^Department of Rehabilitation Medicine, Huashan Hospital, Fudan University, WuLuMuQi Middle Road 12, Shanghai 200040, China; ^2^Department of Neurology, Renji Hospital, School of Medicine, Shanghai Jiao Tong University, Shanghai 200025, China

## Abstract

Poststroke cognitive impairment severely affects the long-term recovery of patients. However, it remains unknown whether an enriched environment can remodel contralateral hippocampal function and promote cognitive function recovery after cerebral ischemic injury. To further explore, 36 C57BL/6 mice that underwent permanent middle cerebral artery occlusion (pMCAO) were randomly assigned to three groups: enriched environment (EE), standard condition (SC), and sham surgery (Sham). After 21 days of intervention, the Morris water maze and step-through test was utilized for testing the cognitive function of the mice, cresyl violet staining for measuring the degree of atrophy in the hippocampal tissues, and western blotting for quantitating the expression levels of GA1B, GAD67, and NR2B, and immunohistochemistry for levels of NR2B in the CA1 region of the contralateral hippocampus. The results showed that cognitive function-related behavioral performance decreased in the SC group, and performance was better in the EE group than that in the SC group (*p* < 0.01); no significant difference in the degree of contralateral cerebral atrophy was observed between the EE and SC groups (*p* > 0.05); levels of GA1B, GAD67, and NR2B in the contralateral hippocampus were significantly higher in the EE group than those in the SC group (*p* < 0.01); and the level of NR2B in the CA1 region of the contralateral hippocampus significantly increased in the EE group compared to the SC group (*p* < 0.01). We believe that contralateral hippocampal function is inhibited after cerebral ischemic injury, further affecting cognitive function. However, enriched environment can upregulate GABAergic and glutamatergic systems in the contralateral hippocampus to promote cognitive function recovery after cerebral ischemic injury.

## 1. Introduction

Cognitive impairment is one of the most common complications of stroke [1]. The long-term effects of poststroke cognitive impairment is much more severe than those of physical impairment and can cause patients to experience difficulties in connection with the perception and adaptation to the external environment. Simultaneously, the cognitive impairment caused by stroke further aggravates other functional deficits. These impairments will ultimately decrease self-care ability, work ability, social function, and psychological health. These defects could aggravate the burden on patients, families, and society [2]. Unfortunately, the benefits of drug treatment on poststroke cognitive impairment are minimal [2, 3]. Therefore, identifying feasible rehabilitation measures and elucidating their effector mechanisms are imperative.

Enriched environment (EE) refers to an intervention of providing equipment and tissue stimulation environment to promote exercise, cognitive activities, and social activities [4]. EE intervention is a new, simple, and effective treatment that is widely used in medical practice, including rehabilitation for cognitive impairment. EE not only provides sufficient multisensory stimulation but also includes training and learning opportunities for social interactions, spatial exploration, and spontaneous exercise activity. Through EE, the opportunities for humans and animals to obtain material and social stimulation from the environment significantly increase. Therefore, EE may play an important role in improving brain plasticity and behavior [5].

Currently, the mechanisms by which EE improves cognitive function are still not completely understood. A previous study found that EE's role in the improvement of cognitive impairment may be associated with restoration of hippocampal neuronal regeneration, increasing the length of myelinated nerve fibers in the hippocampus, or promoting cerebral blood vessels and blood flow in areas around ischemic cortical regions. After mice were exposed to EE for a certain period of time, the volume of cortical neuronal cell bodies and the quantity and length of dendrites increased [6]. EE demonstrated some benefits in various animal models of brain diseases, including improving cognition, delaying disease progression, increasing cell plasticity, and expression levels of related proteins [7].

However, most previous studies on the effector mechanisms of EE on poststroke cognitive impairment focused on the site of brain injury and the area surrounding the infarct [7, 8], and studies on the fundamental mechanisms of indirect injury sites are limited. In this study, we selected a suitable mouse model of permanent middle cerebral artery occlusion (pMCAO). We assumed that contralateral hippocampal GABAergic and glutamatergic systems-related proteins GAD67 (glutamic acid decarboxylase-67), GA1B (GABAB receptor 1), and NR2B (N-methyl-D-aspartate receptor 2B) were upregulated by EE to further reveal the effects and mechanisms by which EE can improve cognitive function after cerebral ischemia [9–11].

## 2. Materials and Methods

### 2.1. Experimental Animals and Experiment Design

**Figure 1:**
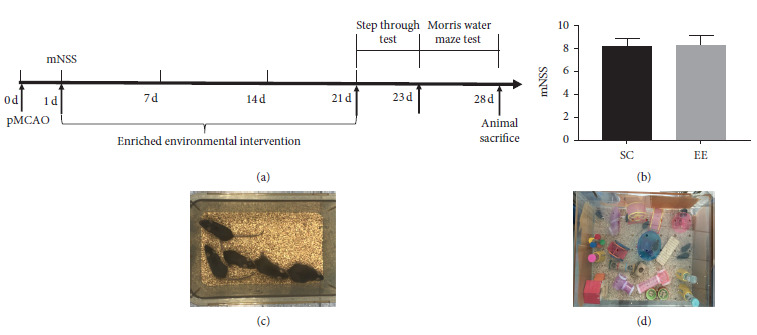
(a) Experiment design. (b) Modified neurological severity scores (mNSS) used at 24 hours after the pMCAO model was constructed. There was no difference between SC and EE groups (*p* = NS, Student's *t*-test). Data are shown as mean ± SD, *n* = 12 per group. (c) Standard condition. (d) Enriched environment. mNSS, modified neurological severity scores; SC, standard condition; EE, enriched environment.

Specific pathogen-free (SPF)-grade, 2 to 3-month-old male C57BL/6 mice weighing 25–27 grams were obtained from Lingchang Biotechnology (Shanghai, China). The mice were reared at the MED-X Research Institute, School of Biomedical Engineering, and Shanghai Jiao Tong University. Before surgery, the mice were distributed to different standard condition cages (5 mice per cage) which were 30 cm long, 20 cm wide, and 15 cm tall. The base of each cage contained 2 cm of bedding material (Figure 1(c)) and given ad libitum access to food and water, daily light cycle of 12 h, and room temperature of 24 ± 1°C. The mice were fasted for 12 h before surgery but allowed to drink water. The experiment protocol was approved by the Institutional Animal Care and Use Committee of Fudan University (approval no. 20160858A232).

A total of 36 mice were used in the experiment, which included 12 mice that received standard environment intervention after the sham surgery (Sham) group and 24 mice that underwent successful pMCAO model construction and were randomized into the standard condition (SC, *n* = 12) group and enriched environment (EE, *n* = 12) group. Twenty-four hours after surgery, various groups of mice were placed in their corresponding environments for rehabilitation intervention.  Figure 1(a) shows the overall study design.

### 2.2. Enriched Environment Intervention

The EE cage was 90 cm long, 70 cm wide, and 40 cm tall. The base of each cage contained 2 cm of bedding material. Twelve mice were kept in the EE cage. The cage contained abundant material stimuli such as slopes, small wooden ladders, platforms for climbing, tunnels, dark boxes, blocks of different colors and shapes, swings for playing and rolling, and treadmills for autonomous exercise. The position of the objects was changed once every three days, and the object combination was changed once a week to maintain novelty (Figure 1(d)). Mice in the SC and Sham groups were returned to standard condition cages.

### 2.3. Preparation of Permanent Middle Cerebral Artery Occlusion (pMCAO) in C57BL/6 Mice

Before the mouse model construction, the mice were housed in standard environments for seven days of acclimatization. pMCAO was performed as previously described [12]. Briefly, a 15 mm long 6-0 nylon suture was used to create the occlusion. The thread occlusion end underwent blunt electrocoagulation, and silica gel was applied to it. The silica gel head length was 1.5–2 mm. Before surgery, 5% of isoflurane was used for anesthesia induction. During surgery, 1.8–2.0% of isoflurane was used for anesthesia maintenance in mice. After anesthesia, a laser Doppler flowmetry (Moor Instruments, Devon, UK) probe was placed vertically on the skull surface for 5 seconds for continuous measurement. After the readings had stabilized, the baseline of cerebral blood flow was recorded. Subsequently, the mice were fixed in a supine position on a 37°C ± 0.5 heating pad. After the skin on the neck was disinfected, a midline incision was made, and the left common carotid artery (CCA), internal carotid artery (ICA), and external carotid artery (ECA) were separated. The external carotid artery and CCA were ligated. An incision was made at the ligation site on the CCA, and a thread occlusion was finally sent through this incision to the middle cerebral artery. Cerebral blood flow was again measured and ensured it was 20 ± 3% of the base line. The thread occlusion was advanced 60–65 mm at the junction of the ICA and ECA. In the Sham mice, dissection of the arteries was conducted except for the insertion of sutures.

### 2.4. Modified Neurological Severity Scores (mNSS)

Approximately 24 hours after the pMCAO model was constructed, mNSS were used for preliminary assessment of the degree of injury in the different groups of mice. The mNSS ranged from 0–14 points and was used to assess the motor, sensory, balance, and reflex functions of the mice [13]. In this study, mouse models with mNSS of 7–9 points were used to simulate cerebral ischemic injury (Figure 1(b)).

### 2.5. Step-through Test

The step-through test is used to measure memory function in animals after stroke [14]. The smartcage system (Precisionary, NC, USA) was employed in this experiment. This system includes an infrared detection base, an electrostimulation base, a red dark box, and a transparent casing. The first day of the test is the training day in which animals were placed in the test cage for 3 minutes of acclimatization. The electrostimulation device at the base of the dark box was then turned on. When a mouse enters the dark box, an electrical stimulus was released one second later. The animal will rapidly escape from the dark box to the light box due to pain. The dark box was powered up for five continuous minutes to strengthen memory. The second day is the test day. After 24 hours of training, mice were placed in the light box with their backs facing the dark box, and the electrostimulation device was turned off. The entire process was recorded for 300 seconds. The time taken for the mouse to first enter the dark box was recorded as step-through latency. The escape latency of mice which did not enter the dark box for the entire experiment was recorded as 300 seconds. In addition, the time the mouse remained in the dark box was recorded.

### 2.6. Morris Water Maze Test (MWM)

The MWM is a common cognitive and behavioral science tool that is used to assess spatial learning and memory [15]. This maze consists of a circular pool that is filled with opaque water. Animals are required to swim toward a submerged circular platform and climb to escape the water maze. The diameter of the maze pool was 122 cm. The height of the pool was 51 cm, water temperature was adjusted to 19–22°C, and the diameter of platform was 10 cm. White titanium oxide was added to the water surface to conceal the position of the platform.

In the six-day period of MWM, positioning navigation test was used for the first 5 days. A pool was divided into four quadrants, namely, southwest, northwest, northeast, and southeast. Also, the underwater escape platform was located at the northeast quadrant. For each day, mice were placed into the water by facing the pool wall at four different entry-points away from the platform. For any mice that were unable to find the platform in 60 seconds, they will be manually guided to the platform. Regardless of how the mice reached the platform, they would be allowed to stay on it for 10 seconds to strengthen learning. At Day 6, the spatial exploration test was initiated. The platform was removed, and the animals were only placed into the water at the opposite quadrant of the platform, southwest. The test duration was 60 seconds.

In the positioning navigation test, escape latency (the time cost to find the platform) was recorded for Days 3–5. Also, in the spatial exploration test, time spent in the northeast zone was recorded. These data were used to evaluate the experimental subjects' ability of spatial learning and memory.

### 2.7. Immunohistofluorescence

After the behavioral science tests, six mice were randomly selected from each group for immunohistochemical staining. Briefly, mice were successively perfused with 0.9% physiological saline and 4% paraformaldehyde (pH 7.4) before entire brain tissues were extracted. A cryomicrotome was used to extract 20 *μ*m-thick coronal cryosections which were then mounted on microscope slides. The sections were fixed in methanol at −20°C for 10 min. Then, diluted donkey serum (Jackson Immuno Research, West Grove, PA) was used for blocking at room temperature for 60 min. The glass slides were incubated with the primary antibody against NR2B (1:200, Proteintech, IL, USA) at 4°C overnight. The next day, the slides were washed with PBS and then incubated with fluorescent secondary antibodies at room temperature for 1 hour. A fluorescence confocal microscope (Leica, Wetzlar, Germany) was used to image the tissue sections. The same parameters were used for taking microscopic photographs. All photographs were captured under identical conditions.

### 2.8. Measurement of Cerebral Atrophy Volume

A series of 20 *μ*m-thick coronal sections were made at 200 *μ*m intervals from the anterior commissure to the hippocampus for measurement of cerebral atrophy volume. The sections were stained with cresyl violet. The ImageJ software (National Institutes of Health, MD, USA) was used to plot and calculate the volume of the ipsilateral and contralateral brain slices. Infarct volume was calculated as described previously [16].

### 2.9. Western Blot Analysis

After the behavior tests, the contralateral hippocampal tissues from the six remaining mice in every group were collected for western blotting. Briefly, the tissues were placed in RIPA lysis buffer containing protease inhibitors and homogenized. After centrifugation, the supernatant was collected. The BSA assay was used to quantitate protein concentration in the supernatant. Protein samples were denatured at 95°C for 10 min. Then, 20 *μ*g were used for SDS-PAGE and transferred onto a nitrocellulose (NC) membrane. The membrane was incubated with blocking solution at room temperature for one hour. Then, the membrane was incubated with primary antibodies at 4°C overnight: GAD76 (1:500, Proteintech, IL, USA), GA1B (1:1000, Abcam, MA, USA), and N-methyl-D-aspartate receptor 2B (NR2B) (1:500, Proteintech, IL, USA). The membrane was then washed and then incubated with horseradish peroxidase-(HRP-)-conjugated secondary antibodies at room temperature for 1 hour. An enhanced chemiluminescence (ECL) reagent kit was used for detection of western blotting results. A western blotting imaging system (Bio-Rad, Hercules, CA) was used to measure the fluorescence intensity of protein bands, and ImageJ was used for quantitation.

### 2.10. Statistical Analysis

All results were presented as mean ± SD. Data were analyzed using SPSS 22.0 software. Student's *t* test or one or two-way repeated ANOVA with the Student–Newman–Keuls multiple comparison test was used in the research. *p* values <0.05 were considered to be statistically significant.

## 3. Results

### 3.1. mNSS in Mice from the SC and EE Groups before EE Intervention

The mice were randomized into the SC and EE groups one day after the pMCAO models were constructed. mNSS of the two groups of mice were assessed for any differences, and there is no significant difference between the mice from the SC and EE groups (*p* = NS) (Figure 1(b)). This shows that the nerve damage caused by the pMCAO model was consistent. Therefore, interference on experimental results after grouping was eliminated, and the experimental results could be more accurate.

### 3.2. Morris Water Maze Performance of Different Groups after EE Intervention

**Figure 2:**
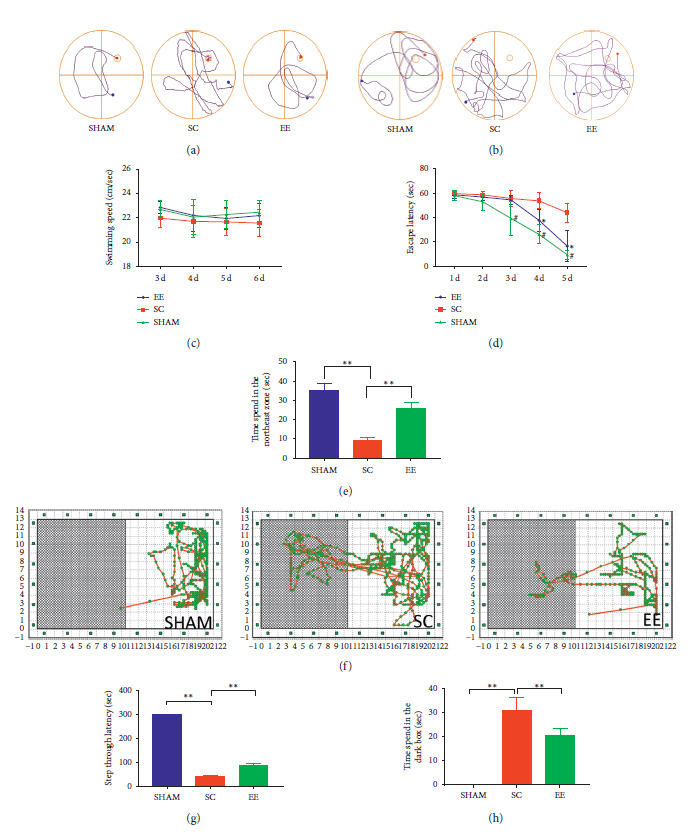
Cognitive function analyses using the Morris water maze and step-through tests. (a) The typical swimming paths of sham, SC, EE groups in positioning navigation test. (b) The typical swimming paths of sham, SC, EE groups in spatial exploration test. (c) The average swimming speed of mice during the last 4 days. No significant difference was shown among these groups (*p* = NS). (d) Escape latency to find the hidden platform for day 3–5. Comparison of the SC and the Sham groups indicated that the former had a longer escape latency in the tests at the last three days (#*p* < 0.05), and the EE group was compared to the SC group, the former showed a shorter escape latency only at the last two days (*∗p* < 0.05). (e) Spatial exploration test, the time spent in the quadrant where the platform was located was longer in both EE and Sham groups compared to the SC group (both *∗∗p* < 0.01). (f) The typical movement paths of sham, SC, EE groups in step-through test. (g) The step-through latency was longer in the EE and Sham groups compared to the SC group (both *∗∗p* < 0.01). (h) The duration in the dark box was shorter in the EE and Sham group. SC, standard condition; EE, enriched environment.

Comparison of the SC and the Sham groups indicated that the former had a longer escape latency in the positioning navigation test at the last three days (*p* < 0.01), and the SC group showed poorer cognitive function. When the EE group was compared to the SC group, the former showed a shorter escape latency only at the last two days (*p* < 0.01) (Figures 2(a) and 2(d)). In the spatial exploration test, the time spent in the quadrant where the platform was located was longer in both EE and Sham groups compared to the SC group (both *p* < 0.01) (Figures 2(b) and 2(e)). This shows that the SC group had poorer cognitive function. From both tests, we can conclude that EE intervention can improve cognitive function after cerebral ischemic injury.

### 3.3. Step-through Test Performance of Various Groups after EE Intervention

The time spent before entering the dark box was longer in the EE group and Sham group compared to the SC group in the step-through test (both *p* < 0.01) (Figure 2(g)), while the duration in the dark box was shorter in the EE and Sham groups compared to the SC group (both *p* < 0.01) (Figures 2(f) and 2(h)). This again shows that the SC group had poorer cognitive function and EE intervention that can improve cognitive function after cerebral ischemic injury.

### 3.4. Cresyl Violet Staining and Calculation of Atrophy Volume in Hippocampal Brain Slices

**Figure 3:**
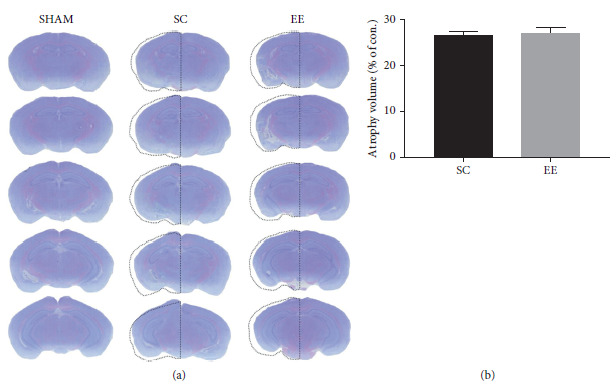
Measurement of cerebral atrophy volume (a) Photographs showed brain coronal sections with cresyl violet staining in the sham, SC and EE groups. (b) No statistical difference in cerebral atrophy volume between the SC and EE groups (*p* = NS). Data are shown as mean ± SD, *n* = 6 per group. *p* = NS. SC, standard condition; EE, enriched environment.

There is no statistical difference in the relative cerebral atrophy volume (% of con.) between the EE and the SC groups (*p* = NS) (Figure 3). EE cannot reverse the incurred damage to the structure of the hippocampus in the pMCAO model.

### 3.5. Improvement Status of GABAergic Nervous System in Contralateral Hippocampus of Various Groups after EE Intervention

**Figure 4:**
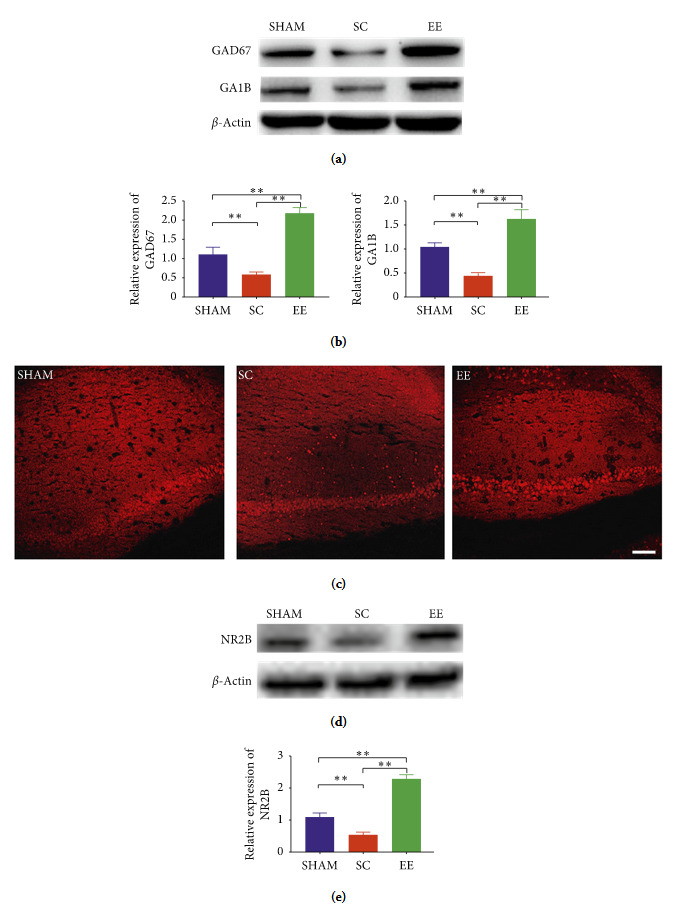
GABAergic and glutamatergic systems in the contralateral hippocampus of cerebral ischemic injury were inhibited. EE can upregulate the aforementioned systems related protiens. (a) The expression levels of GAD67 and GA1B proteins that are related to the GABAergic nervous system were higher in the EE and Sham groups compared to the SC group (both *∗∗p* < 0.01). (b, c) Immunofluorescence and Western blot analysis demonstrated that the expression of NR2B related to the glutamatergic systems significantly reduced in the SC group compared to the sham or EE groups (both *∗∗p* < 0.01). Scale bar = 100 *µ*m. Data are shown as mean ± SD, *n* = 6 per group. SC, standard condition; EE, enriched environment; GAD76, glutamic acid decarboxylase-67; GA1B, GABAB receptor 1; NR2B, N-methyl-D-aspartate receptor 2B.

The expression levels of GAD67 and GA1B proteins that are related to the GABAergic nervous system were higher in the EE and Sham groups compared to the SC group (both *p* < 0.01) (Figures 4(a) and 4(b)). This indicated a deterioration of GABAergic neurological function in the contralateral hippocampus during the chronic phase of cerebral ischemic injury. However, EE intervention can upregulate the expression of GAD67 and GA1B.

### 3.6. Expression of Glutamatergic Receptors in Contralateral Hippocampi of Various Groups after EE Intervention

Immunofluorescence and western blot analysis demonstrated that the expression of NR2B related to the glutamatergic systems significantly reduced in the SC group compared to the sham groups (*p* < 0.01). EE intervention can reverse this phenomenon (Figures 4(c)–4(e)).

## 4. Discussion

In this study, we found that the pMCAO model may cause persistent cognitive impairment, and both GABAergic nervous system and glutamatergic receptors in the contralateral hippocampus of cerebral ischemic injury were inhibited. EE promotes recovery of cognitive function after cerebral ischemic injury that may in part be mediated by upregulating GA1B, GAD67, and NR2B which were relevant to the aforementioned systems in the contralateral hippocampus.

The MCAO model is widely used in basic research on stroke, and common models include transient middle cerebral artery occlusion (tMCAO) and pMCAO [17]. The core difference between these two models is whether thread occlusion was moved. In clinical practice, patients with central nervous system damage, particularly those with chronic phase cerebral ischemic injury, mainly undergo neurological rehabilitation. These patients usually miss the opportunity for acute thrombectomy and thrombolysis, resulting in an inability for spontaneous recovery of neurological function for a long period of time [18]. Therefore, we selected the pMCAO model, which is similar to the clinical problems described in this study. In our present study, there is no significant difference in mNSS after pMCAO surgery was observed between the SC and EE groups, which excluded the effects of differences in degree of injury before grouping.

Cresyl violet staining used in the pMCAO model indicated that there was no statistical difference in cerebral atrophy volume between the SC and EE groups. This indicates that when permanent ischemic injury occurs in the middle cerebral arteries, EE cannot reverse the incurred damage to the structure of the hippocampus in the MCAO model. The hippocampus plays an important role in information processing, memory formation, and subsequent behavioral regulation [19]. In the Morris water maze and step-through tests, which are highly correlated with the cognitive function of the hippocampus, mice from the SC group showed a significant decline in cognitive function compared to the Sham group. This shows that cognitive impairment caused by the pMCAO model persists. However, EE intervention could improve cognitive impairment after cerebral ischemic injury. This further suggests that EE may participate in compensatory functions in the contralateral hippocampus.

Excitation/inhibition balance is mainly maintained by the GABAergic nervous system in the central nervous system [20]. In our present study, the experiment results first showed that compared to mice from the Sham group, the expression of GAD67 and GA1B in the contralateral hippocampi of SC mice was downregulated, indicating a decline in the GABAergic nervous system of the contralateral hippocampus during the chronic phase of cerebral ischemic injury. GABA is synthesized by GAD67 and GAD65. In the central nervous system, GAD67 plays an important role in the synthesis and synaptic release of GABA [9]. Further, GABA can activate GABA receptors that are located in various neurons in the brain. In particular, reduction in GABA B receptors during cerebral ischemia has been observed [21]. The activation of GABA B receptors can restore the balance in the cell membrane surface expression of cyclic-nucleotide-gated cation nonselective (HCN)1/HCN2 in the CA1 region in the rat hippocampus, and the HCN1 channel regulates distal synaptic input to integrate dendrites in the pyramidal cells to participate in learning and memory [22]. In addition, a recent study reported that GA1B is an effective factor that improves cognitive impairment caused by chronic insufficient cerebral perfusion [11]. Therefore, during cerebral ischemia injury, the upregulation of the GABAergic nervous system (such as GAD67 and GA1B) in the hippocampus may improve cognitive impairment. Previous study mainly focus on the ipsilateral hippocampus after cerebral ischemia. However, our study suggested EE upregulated the expression of GAD67 and GA1B in the contralateral hippocampus and could promote recovery of cognitive function.

Ischemic stroke triggers the transient efflux of large levels of glutamate and overactivation of NMDAR, which may lead to neuronal death [23, 24]. The results of this study showed that NR2B expression decreased during the chronic phase in the pMCAO model and showed for the first time that EE increased NR2B expression and enhanced synapse plasticity in the contralateral hippocampus, which may promote recovery of cognitive function. We also found that the protein expression in EE is higher when compared with SHAM. The traditional viewpoint is that NMDAR, particularly NR2B, accelerates neuronal death during cerebral ischemic injury and should be inhibited. However, these studies involved observations during the acute phase of stroke, but few studies have evaluated the chronic phase. Therefore, most clinical trials on NMDA antagonists cannot improve and may even worsen stroke outcomes [10, 25]. A recent study revealed that the persistent decline in NMDAR density after stroke plays an important role in plasticity and memory formation. This shows that NMDAR activity should be promoted instead of inhibited during the nonacute phase of stroke. NMDAR excitation during the poststroke recovery stage may have beneficial effects to the cognitive function, which may be due to enhanced neuroplasticity and not neuroprotective effects [26].

Our prediction is that the ipsilateral hippocampus after permanent cerebral ischemic injury can no longer be responsible for cognitive function. Thus, contralateral hippocampus must be step up and compensate for this cognitive function. This leads to the increase of related protein in the contralateral hippocampus to achieve the desired outcome. In clinical practice, the most common method for direct regulation of brain excitation is repetitive transcranial magnetic stimulation (rTMS). The traditional viewpoint is that excitatory stimulation should be used on the ipsilateral cortex, whereas inhibitory stimulation should be used on the contralateral cortex during poststroke rehabilitation. [27] However, recent studies have shown that excitation of the contralateral cortex can improve motor function in patients when there is severe damage to the ipsilateral cortex after stroke, and motor function shows persistent signs of no recovery [28]. This study may have similar concepts as the aforementioned recent studies.

In summary, contralateral hippocampal function is inhibited after cerebral ischemic injury, further affecting cognitive function for a prolonged period. However, enriched environment can upregulate GABAergic and glutamatergic systems in the contralateral hippocampus to promote cognitive function recovery. However, this speculation needs further investigation and requires further clinical trials for validation.

## Data Availability

The data used to support the findings of this study are available from the corresponding author upon request.
